# Emotion-aware music tower blocks (EmoMTB ): an intelligent audiovisual interface for music discovery and recommendation

**DOI:** 10.1007/s13735-023-00275-8

**Published:** 2023-06-02

**Authors:** Alessandro B. Melchiorre, David Penz, Christian Ganhör, Oleg Lesota, Vasco Fragoso, Florian Fritzl, Emilia Parada-Cabaleiro, Franz Schubert, Markus Schedl

**Affiliations:** 1 Institute of Computational Perception, Johannes Kepler University Linz, Linz, Austria; 2 Human-centered Artificial Intelligence, Linz Institute of Technology, Linz, Austria; 3 TU Wien, Vienna, Austria; 4 Salzburg University of Applied Sciences, Salzburg, Austria; 5 University of Applied Arts Vienna, Vienna, Austria; 6 St. Pölten UAS, St. Pölten, Austria

**Keywords:** Intelligent user interface, Music discovery and exploration, Affective computing, Emotion recognition, Clustering, Recommender system

## Abstract

Music listening has experienced a sharp increase during the last decade thanks to music streaming and recommendation services. While they offer text-based search functionality and provide recommendation lists of remarkable utility, their typical mode of interaction is unidimensional, i.e., they provide lists of consecutive tracks, which are commonly inspected in sequential order by the user. The user experience with such systems is heavily affected by cognition biases (e.g., position bias, human tendency to pay more attention to first positions of ordered lists) as well as algorithmic biases (e.g., popularity bias, the tendency of recommender systems to overrepresent popular items). This may cause dissatisfaction among the users by disabling them to find novel music to enjoy. In light of such systems and biases, we propose an intelligent audiovisual music exploration system named EmoMTB . It allows the user to browse the entirety of a given collection in a free nonlinear fashion. The navigation is assisted by a set of personalized emotion-aware recommendations, which serve as starting points for the exploration experience. EmoMTB  adopts the metaphor of a city, in which each track (visualized as a colored cube) represents one floor of a building. Highly similar tracks are located in the same building; moderately similar ones form neighborhoods that mostly correspond to genres. Tracks situated between distinct neighborhoods create a gradual transition between genres. Users can navigate this music city using their smartphones as control devices. They can explore districts of well-known music or decide to leave their comfort zone. In addition, EmoMTB   integrates an emotion-aware music recommendation system that re-ranks the list of suggested starting points for exploration according to the user’s self-identified emotion or the collective emotion expressed in EmoMTB ’s Twitter channel. Evaluation of EmoMTB   has been carried out in a threefold way: by quantifying the homogeneity of the clustering underlying the construction of the city, by measuring the accuracy of the emotion predictor, and by carrying out a web-based survey composed of open questions to obtain qualitative feedback from users.

## Motivation and background

**Fig. 1:**
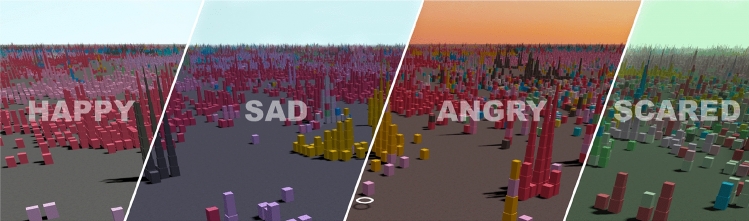
EmoMTB ’s  landscape for the four emotional themes

Listening to music is an essential part of human life. Over the last decade, digital music streaming platforms have become the predominant way of music consumption. Service providers such as Spotify, Deezer, or Amazon Music offer their users access to tens of millions of recordings.^1^$$^{,}$$^2^$$^{,}$$^3^ To help users manage such huge collections and identify music that suits their tastes, streaming platforms commonly offer text-based search and recommendation functionalities. The former provides an efficient means to find a particular artist, album, or track whose name is known by the user. The latter recommend lists of tracks tailored to the music listener. Such lists are confined, however, by their unidimensional structure, even though major music recommendation platforms recently introduced mechanisms such as shelves, channels, or carousels to provide additional—again linearly organized—lists of music tracks.

While they constitute an effective and widely adopted way to present retrieval and recommendation results, list representations bear a number of shortcomings. Due to natural cognition biases, users can only effectively interact with lists of limited lengths and even with such lists they tend to pay more attention to items situated on the first positions [1, 2]. This, combined with biases often present in recommendation algorithms [3, 4], results in a considerable portion of potentially valuable items that are never presented to the user.

One possibility to transcend this unidimensional linear way of interacting with music collections is clustering-based audiovisual interfaces [5, 6]. They empower users to explore large music collections in a nonlinear way, taking advantage of vision being the human sense with the largest information bandwidth. This paper presents such a novel audiovisual user interface, which we refer to as *Emotion-aware Music Tower Blocks* (EmoMTB ).

EmoMTB   adopts the metaphor of a city to allow for the navigation of large music collections. The city is composed of many buildings (tower blocks), each of which comprises several cubes. Each cube represents a single music track. Figure 1 depicts the general appearance of the interface. The layout of the city, i.e., the position of blocks and buildings, is determined by an underlying dimensionality reduction algorithm that identifies clusters of tracks that share similar audio properties and genres. Therefore, highly similar songs can be found in the same building. Nearby buildings form districts of a certain genre. Cubes are colored to distinguish their genres. Adopting this strategy, EmoMTB   enables users to explore the music collection either within their comfort zone (staying in regions of their preferred genres) or outside of it (leaving well-known genre neighborhoods). Users can navigate in the city using their smartphone as a control device. Also, they may request recommendations based on their music preferences and can explore them directly in the landscape. In addition to such recommendations that are solely personalized to their music taste, users are provided with recommended tracks that match their emotional state. To this end, EmoMTB   adopts emotion recognition techniques to classify each track into one of four affective categories based on its user-generated tags gathered from the music platform Last.fm. Based on this classification, recommendation lists are then tailored to the user’s self-identified emotional state or to the emotion predicted from postings to EmoMTB ’s  Twitter channel.

The main contribution of the paper is a working prototype of EmoMTB ,  a novel audiovisual interface allowing for free music browsing, assisted by an emotion-aware recommendation system. EmoMTB   provides a unique experience due to the set of the following features, which, to the best of our knowledge, do not appear in this combination in any other system:

- EmoMTB   integrates tracks from LFM-2b [7, 8], a recent large-scale dataset, allowing to cluster and present to users a collection of almost half a million music tracks. This number substantially exceeds collections supported by previous audiovisual music exploration interfaces [9–12].
- Due to the track projection and clustering approach that takes into account both audio and genre features, music tracks in EmoMTB   are placed in a 2-dimensional space that enables smooth music genre transition. This means that the user can employ their sense of direction to navigate towards tracks they are more likely to enjoy.
- EmoMTB   provides each user with a set of personalized emotion-aware recommendations, serving as starting points for the exploration. This allows users to fully benefit from the clustered layout of the tracks and find new enjoyable tracks quicker. Therefore, users can start from a recommended track that matches their taste and then investigate nearby tracks, finely steering from music at the core of their preferences towards more diverse music.

The remainder of the paper is organized as follows: Section 2 reviews research on related music discovery interfaces, music emotion recognition, and emotion-aware music recommendation. Section 3 describes EmoMTB ’s interface and functionalities for user interaction. Subsequently, Sect. 4 details the methods adopted to create the different parts of EmoMTB   and realize its functionalities. As for evaluation, Sect. 5 elaborates on the three experiments we conducted to assess the quality of EmoMTB ’s  various components. Finally, Sect. 6 rounds off the work with a summary and a discussion of limitations and future avenues.

## Related work

The work at hand is embedded into the research areas of intelligent music exploration interfaces, music emotion recognition, and emotion-aware music recommendation.

### Music exploration interfaces

Existing user interfaces that foster interactive audiovisual exploration of music collections commonly create a spatial arrangement of the discoverable music pieces. Early systems include *Islands of Music* [5], *nepTune* [13], and *deepTune* [9]. These interfaces organize the music tracks of a collection according to their audio features, where similar tracks are clustered to form ‘islands’ (dense regions) that raise from the ocean (sparse regions), adopting the metaphor of a geographic landscape. In a similar fashion, *Music Galaxy* [14] visualizes a music collection adopting the metaphor of the universe. The positioning of the stars, representing music tracks, is determined by a distance metric computed over audio features. Stars can also be rearranged and adapted to the taste of the user. The metaphor of a planetarium is used in *Songrium* [11], which is a web-based application to facilitate interactive exploration of music on video streaming platforms. Songrium applies similarity-preserving projection techniques to map songs to galaxies, based on audio and web content. It offers its users various perspectives of the galaxy and enables them to explore derivative music works. More recently, Shen et al. [15] propose *MusicLatentVIS*, a tool to investigate and explore collections of traditional Chinese music. For this purpose, music feature representations are learned via deep learning algorithms (in particular autoencoders), whose latent representations are projected into a 2-dimensional space by applying t-student-distributed stochastic neighborhood embedding (t-SNE) [16]. In the resulting interactive interface, users can bring up additional information visualizations such as parallel coordinates or visualizations of acoustic similarity matrices. Schedl et al. [17] propose an interface that leverages audio features and genre data, and again a t-SNE data projection, to create a skyline landscape the user can navigate. The different parts of the landscape can be colored according to the values of the audio descriptors (e.g., energy) or genres.

There also exist a few music exploration interfaces that incorporate emotion information. For instance, Vad et al. [10] create a t-SNE-based visualization from emotion-related descriptors of songs which they extract from the audio. The user can interact with the visualization and create playlists by drawing lines in the 2-dimensional t-SNE projection. Liang and Willemsen [12] propose an audiovisual interface to discover new music genres based on emotions. They use the energy and valence features retrieved through Spotify’s Audio Features & Analysis API and represent tracks within a contour plot visualization along those two features, which the user can interact with. A more comprehensive survey of intelligent music discovery interfaces is provided by Knees et al. [6].

**Table 1:** Comparison of collection sizes among the reviewed music exploration interfaces

Paper	# of tracks
Islands of Music [5]	359
nepTune [13]	50
deepTune [9]	48.000
Music Galaxy [14]	unspecified
Songrium [11]	100.000
MusicLatentVIS [15]	373
MTB [17] (previous version)	500.000
Vad et al. [10]	20.000
Liang and Willemsen [12]	33.000
EmoMTB	436.064

In comparison with the above works, we highlight EmoMTB ’s  differences below. (1) EmoMTB   creates a large landscape, accessible by the user, which comprises almost half a million tracks. A comparison of EmoMTB   with other similar interfaces with respect to music collection sizes is reported in Table 1. (2) As EmoMTB   directly connects to the streaming service Spotify to play the selected tracks, it does not require the large music collection to be available locally, thus differentiating itself from most of existing interfaces [5, 9, 13]. (3) EmoMTB ’s  landscape is generated by a clustering method that takes into account both audio features and genre information. This allows it to create a space of continuous music genre transitions which makes it easier to explore new music within users’ familiar genres districts, lingering in their comfort zone; or to adventure in new zones of the map of unfamiliar genres, thereby leaving their comfort zone along a semantically meaningful continuum of genres. In contrast, past interfaces [5, 9, 10, 13, 14] mostly consider only audio features. (4) EmoMTB   provides personalized song recommendation lists by providing a connector to Spotify, enabling its users to locate and embed their music taste (profile) within a large music catalog, and to travel to the blocks corresponding to the respective songs in the landscape. Not only does this enable the users to listen to their personalized track recommendations, but also to explore similar tracks in the neighborhood. Existing interfaces, beyond allowing the users to move within the visualization, at most provide text-based search to look for specific tracks within the landscape. (5) The listener’s emotion is considered during the personalization of the recommendation lists and is integrated into the visualization. This aspect is absent from other audiovisual interfaces with the exception of [12], where mood information adjusts the recommendation. (6) The user navigates EmoMTB ’s  visualization through their personal smartphone using a gamepad-like controller instead of the commonly used keyboard and mouse settings [5, 10–12, 14]. The only exceptions are Schedl et al. [9] and Knees et al. [13], which allow the use of a gamepad controller. For many users, in particular smartphone-avid individuals, this represents a more natural way of interaction with apps.

### Music emotion recognition

Music’s ability to express emotions is generally acknowledged [18]. Research in music emotion recognition (MER) typically focuses on extracting emotional content from acoustic cues [19], lyrics [20], codified musical syntax [21], or a combination of the aforementioned ones in a multimodal fashion [22–24]. Nevertheless, despite the advances in MER [25–27], it is still not clear which sources are most reliable to identify users’ perceived emotions. In addition, extracting the aforementioned characteristics requires access to the music audio, which is typically limited by copyright restrictions, which confines (academic) research to experimentation on small- to medium-sized music collections.

With the evolution of social media, a variety of platforms that enable sharing user-generated content related to music consumption and characterization, such as collaborative listening information or tags [28], have emerged. In contrast to other sources, user-generated tags are freely available, thus having a great potential for MER research. Nevertheless, this source, unlike acoustic, symbolic, or lyrical representations, has rarely been used in previous works as a means to detect the underlying emotions in music [29, 30], having been mainly considered for semi-supervised methods to approach MER, e.g., by Wu et al. [31]. Similarly, Panda et al. [32] have also used emotion-related metadata derived from the AllMusic^4^ platform to detect songs’ emotions. However, unlike other user-generated tags, e.g., those from the music social network Last.fm,^5^ AllMusic data is not freely accessible, which impairs the reproducibility of the results and limits further experiments.

### Emotion-aware music recommendation

Integrating emotion information into music recommendation is an emerging research area. For instance, Deng et al. [33] propose a system that recommends music based on emotions and listening information extracted from Sina Weibo, a popular Chinese microblogging platform. The authors adopt a lexicon-based approach to classify emotions from microblogs into up to 21 categories. A mapping between songs and emotions is then created by considering the emotions in microblog messages directly preceding or following a user’s message about music listening. This results in triples of user, song, and emotion vector, i.e., term frequencies over the emotion categories. To recommend songs, the authors adapt user-based and item-based collaborative filtering algorithms as well as a graph-based approach using PageRank. Kaminskas et al. [34] propose a recommender system that suggests music tailored to points-of-interest (PoIs), using an emotion-based matching approach. The authors first conduct crowd-sourced user experiments to obtain annotations for both PoIs and music pieces, based on a list of 20 emotions. To enlarge the music catalog from which recommendations can be drawn, a music auto-tagger is trained on the manual annotations and used to predict missing music emotions. Music recommendations for a given PoI are then created adopting a nearest neighbor approach based on Jaccard similarity between the PoI’s emotion set and the music’s emotion set. Andjelkovic et al. [35] introduce the *MoodPlay* recommendation interface, which integrates audio features and emotion tags into a hybrid music recommendation algorithm. Based on a user-provided artist name, a ranked list of artists is computed and represented within a latent space projection forming a mood space, which the user can explore. Additional recommendations can be brought up based on artists located nearby the center of the user’s artist profile in the mood space, or along the path of the user during navigation in the space.

Different from the previously discussed works, Ayata et al. [36] leverage users’ physiological signals. The authors conceptualize a music recommendation architecture that integrates emotional responses to previously recommended songs. These responses are inferred from various physiological signals acquired from wearable sensors, e.g., for heart rate or skin conductance. Statistical summaries and moments of these signals over time windows are used to predict the user’s valence and arousal. For a more in-depth survey on emotion-aware music recommendation, we refer the reader to Assuncao et al. [37].

## Functionality and interaction

The EmoMTB   interface provides a novel and exciting way to discover new songs while exploring a large music collection (up to half a million of tracks), by engaging the users with two interacting channels: (1) a large monitor that depicts EmoMTB ’s  landscape, the user’s playable avatar, and tracks’ metadata, and (2) the user’s mobile phone for settings and controls to both modify and navigate through the landscape.

**Fig. 2:**
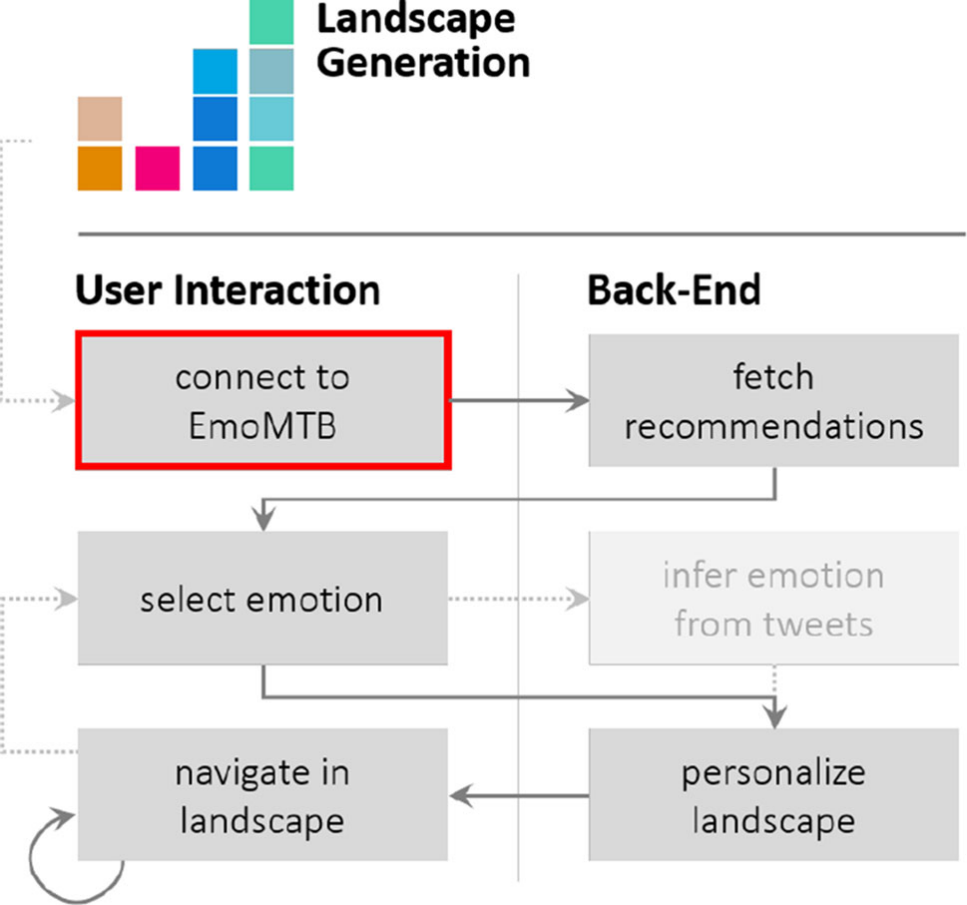
Diagram of EmoMTB ’s  user-system interaction

Initially, the user has to get started by following a setup procedure (see Fig. 2). First, the user connects to EmoMTB   using their mobile phone (Sect. 3.1). As the landscape has been generated prior to this procedure (Sect. 3.2), the user is now able to interact with our application in various ways, e.g., freely explore the landscape at their desire (Sect. 3.3). In addition, EmoMTB   will fetch personalized recommendations (Sect. 3.5), which can further be altered by the user by selecting an emotion (Sect. 3.4). The selected emotion can be changed by the user at any point while exploring the landscape.

### User Onboarding

**Fig. 3:**
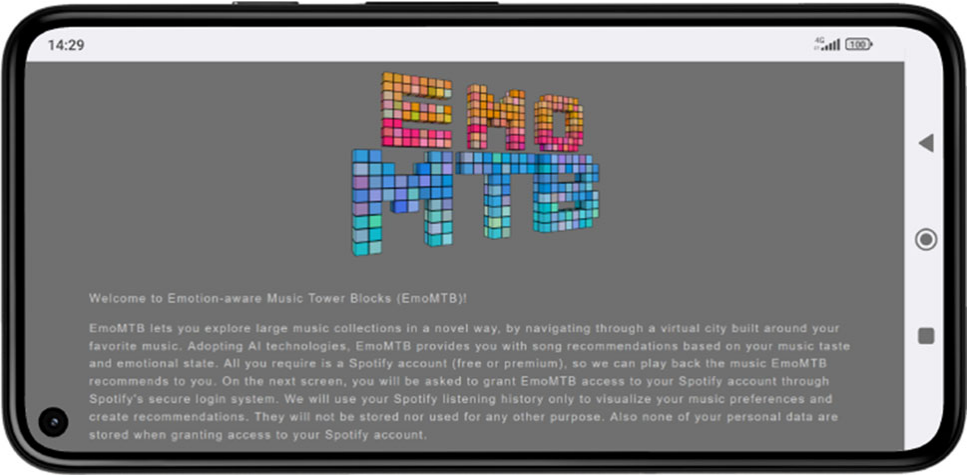
EmoMTB ’s  landing page

As first step for connecting to EmoMTB , the users either scan a QR code with their phone’s camera or manually insert a URL in their browser that, in turn, leads them to the EmoMTB ’s  landing page, depicted in Fig. 3.

After reading a brief explanation and the General Data Protection Regulation (GDPR) notice, the users are asked for their consent for EmoMTB   to fetch Spotify’s listening history data, used to generate the track recommendations. The users can optionally leave their email addresses for further updates about EmoMTB   and participate in a follow-up research study (Sect. 5.3).

### Landscape Appearance

**Fig. 4:**
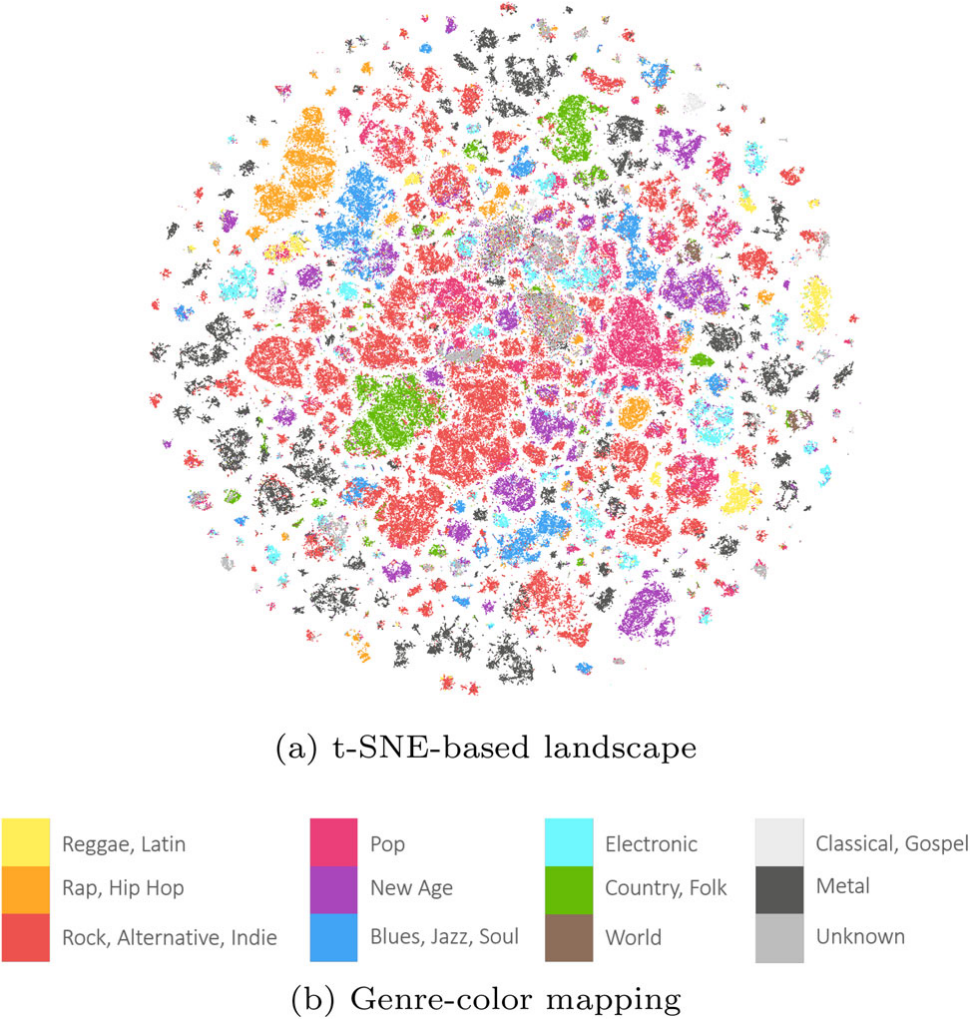
EmoMTB   map

EmoMTB ’s  city-like landscape is comprised of several colorful track-blocks clustered according to their genres and audio features. Highly similar tracks appearing in the same position form towers of blocks, which themselves form neighborhoods of a certain genre. To assist the music exploration of the landscape, we assign a color to each block using its associated fine-grained music genres (Sect. 4) and delineate a genre-color mapping based on the results from the user study presented by Holm et al. [38]. EmoMTB ’s  landscape seen from above and the genre-color mapping are shown in Fig. 4a and b, respectively.

As we can see from Fig. 4a, EmoMTB ’s  world appears segmented in several neighborhoods of different macro-genres, with red and pink being the most numerous. We further notice two aspects about the landscape. First, tracks of the same macro-genre might form different districts, e.g., this is clearly visible for Metal. As we will further detail in Sect. 4.1, EmoMTB ’s  landscape is generated by considering fine-grained music genres. Therefore, even if two tracks belong to the same macro-genre (e.g., Metal), they might appear in different districts depending on their sub-genres (e.g., Trash Metal and Doom Metal). Second, a district might contain tracks belonging to different macro-genres (e.g., Rock+Metal or Pop+Electronic), as the latter might equivalently describe the genre of some tracks (e.g., Rock Metal and Electro Pop tracks). Following these considerations, EmoMTB   enables users to explore new music within their familiar genres districts, lingering in their comfort zone, or to adventure in new zones of the map of unfamiliar genres, thereby leaving their comfort zone.

### Navigation and interaction

**Fig. 5:**
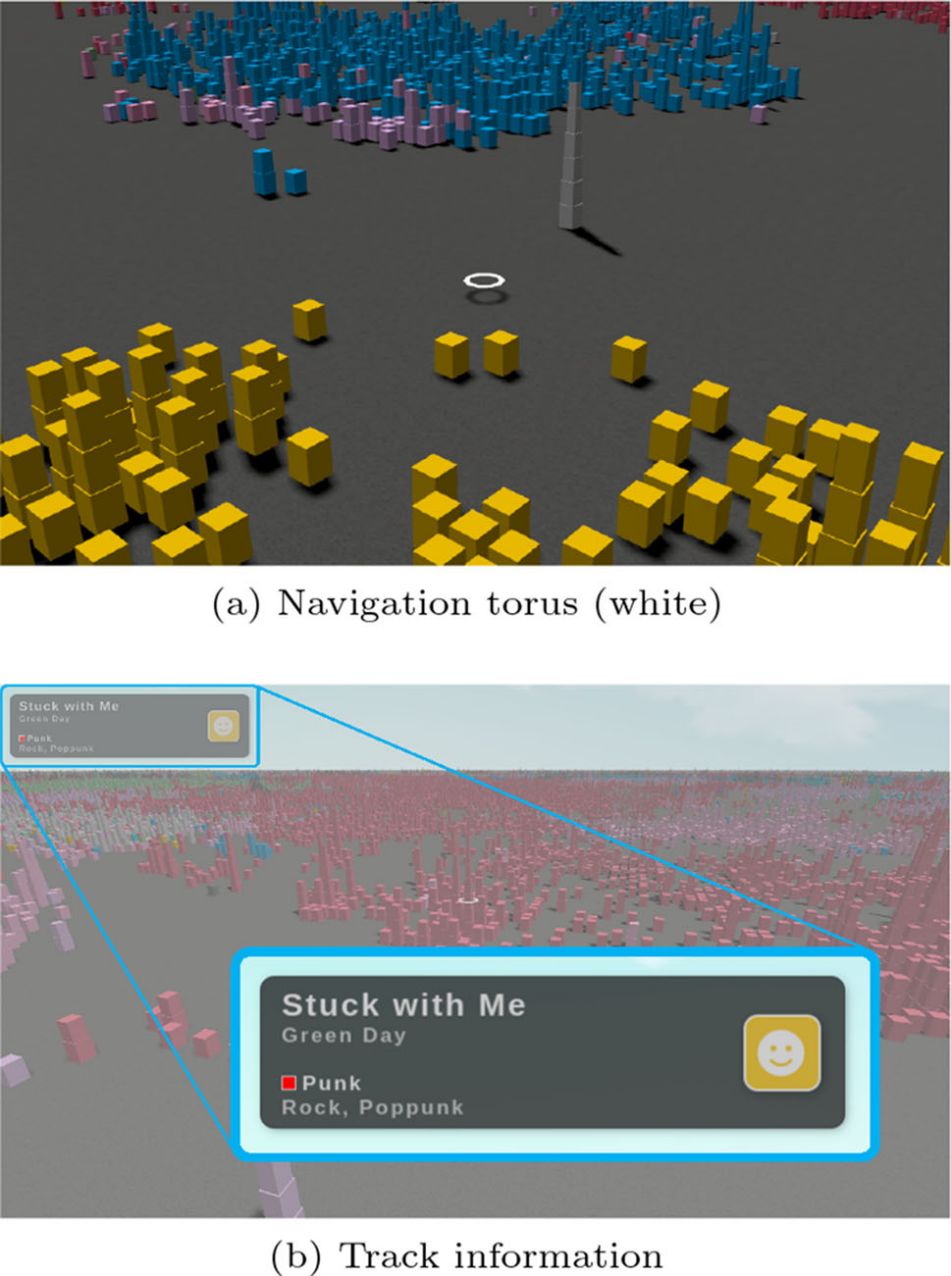
Landscape visualization

**Fig. 6:**
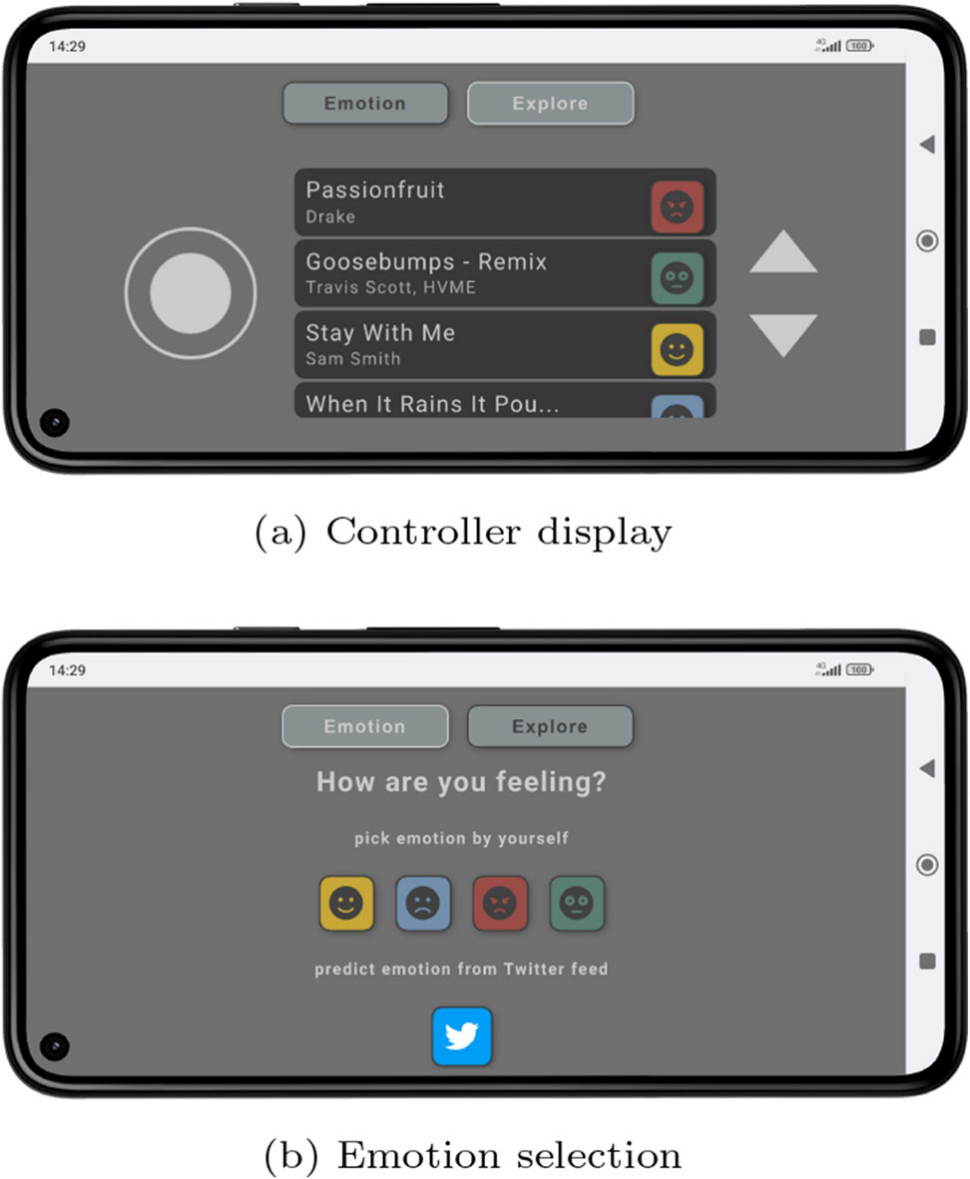
User’s phone interface

To navigate EmoMTB ,  the user controls a playable avatar in the shape of a white hovering torus (see Fig. 5a). The user uses the torus to both explore the landscape and to select blocks by placing it around them. When a block is selected, the track’s name, artist, corresponding fine-grained genres, and predicted emotion (Sect. 3.4) are displayed in the upper left corner of the visualization (see Fig. 5b). If the user stays still over a block for more than 2 s, the playback of its corresponding track starts and continues until the user either hovers over another block for 2 s or stays still on an empty space for 5 s. Such delays in the play and stop of the playback avoid sudden music disruptions and allow users to enjoy the music they picked while still roaming around the landscape.

Moving the white torus can be done with a controller interface specifically designed to run on the browser of the user’s smartphone (see Fig. 6a). A joystick on the left of the display is used to both move the torus over the map and to rotate the visualization. To easily allow the user to navigate the vast map of EmoMTB ,  the controllers are enhanced with linear acceleration, i.e., while the user continuous to move straight ahead, the avatar continuously increases its speed until it reaches a maximum. When the torus is selecting blocks of a tower, the user can use the two arrow buttons shown on the right side of the navigation interface to travel vertically within the building, in analogy of using an elevator. Finally, the user’s personalized recommendations are shown in a scrollable list at the center of the interface, each one displaying the track’s name, artist, and predicted emotion. By pressing on a recommended track, the visualization transports the user’s avatar to the position of the track in the landscape through a smooth animation. Not only does this enable the user to listen to the recommended track but also to explore similar tracks in its neighborhood.

### Emotion selection

The smartphone interface includes a menu where users may manually select one of the four considered emotions, i.e., happiness, sadness, anger, and fear (see Fig. 6b). In addition, another option allows participants to choose an automatically predicted emotion, interpreted as the ‘crowd’s emotion’. This emotion is extracted from the users’ most recent tweets mentioning EmoMTB ’s  Twitter account. The user’s emotional state is then taken into consideration when creating a list of recommended songs (Sect. 3.5), which are also labeled according to one of the four possible emotions.

Besides the recommendation list, users’ selected emotion also alters the landscape by changing the color of the sky and light intensity, thus reflecting better the affective state (see Fig. 1). For example, when ‘happiness’ is selected, the sky turns bright and blue (symbolic of a nice summer day), while ‘fear’ shows an eerie environment, with dimmed lighting.

Lastly, on the right-hand side of the tab that presents the selected track’s information, a song’s emotion is also displayed through an emoji (see Fig. 5b). This emotional information is also shown in the scrollable list with the recommendations, by this helping the user to choose a song to listen to next by taking the track’s underlying emotion into consideration (see Fig. 6a).

### Recommendations

During the onboarding procedure, EmoMTB   retrieves a personalized recommendation list for the user from the Spotify API.^6^ The interface of the user’s smartphone initially displays this full list, minus the tracks that are not part of EmoMTB ’s  catalog (see Fig. 6a). After selecting one of the four emotions (see Sect. 3.4), the list is reordered to show the tracks labeled with the selected emotion on top. As stated in Sect. 3.3, the user can then select individual tracks to move to the corresponding location within the landscape.

## Methodology and implementation

We next detail the procedure we followed to implement the components of the EmoMTB   interface.

EmoMTB   is based on the LFM-2b dataset^7^ [7, 8] which comprises 2 billion listening events of 120 thousands Last.fm users for circa 51 million tracks. Among the available features, the dataset provides metadata and community-assigned tags (e.g., *’rock’*, *’AWESOME’*, *’travel’*) for the music tracks. In particular, each tag is associated with a weight between 0 to 100 that indicates the relative number of users who assigned the tag to the track (e.g., (*’rock’*, 90)). We further augment these track features with audio features and a popularity measure (Sect. 4.1) from the Spotify’s API. To do so, we first query the Spotify API^8^ with the track and artist names of the LFM-2b tracks and retrieve the Spotify URI of the closest result. To ensure an accurate matching between the LFM-2b tracks and Spotify’s catalog, we only match a track if the string similarity, in terms of normalized longest common sequences of characters, between track and artist names is above 0.5 (empirically chosen). We then use the Spotify URIs of the LFM-2b tracks to fetch audio features from Spotify.^9^ Ultimately, we end up with a collection of 436,064 tracks, which we use to build EmoMTB ’s  landscape.

### Landscape generation

In order to create the city-like landscape of EmoMTB ,  we project the tracks of the collection onto a 2-dimensional plane using the widely adopted t-SNE algorithm [16]. As input to the algorithm, each track is represented by both fine-grained genres and audio features. As for the former, we extract each track’s genre information from its Last.fm community-assigned tags by matching them against the extensive EveryNoise^10^ list of micro-genres. This results in 2,374 unique genres covered by the tracks in our music collection. Each track is then represented as a TF-IDF vector adopting as term frequency the Last.fm tag weights and as document frequency the number of tracks sharing the same tag. As for the audio features of the tracks, we use those fetched from Spotify, i.e., *Energy* (intensity and activity), *Valence* (probability of the track conveying positiveness), *Acousticness* (probability that a song is acoustic), *Instrumentalness* (probability of not containing vocals), and *Speechiness* (presence of spoken words). Ultimately, we collect 2,379 features per track (TF-IDF genre weights and audio features).

Before using t-SNE, we apply principle component analysis (PCA) by selecting a number of components (i.e., 405) that covers 95% of the explained variance (empirically chosen), resulting in compacted representations of the tracks. We then use these compact representations as input to the t-SNE (setting perplexity to 45, again empirically chosen), which projects the tracks to a 2-dimensional coordinate space, subsequently discretized to obtain a tiled map.

After this step, the tracks are visualized as colored boxes on the map and tracks that have very similar coordinates are stacked on top of each other while being sorted based on their popularity according to Spotify, with the most popular being on top. This resembles the metaphor of more important people in a company building occupying offices at higher floors. The color of a block is based on the track’s genre. We first identify 12 macro-genres (adapting the genre list investigated in Holm et al. [38]) and then delineate a genre-to-color mapping based on the results of a user study, also carried out by Holm et al. [38]. From the genres associated with a track, we pick the one with the highest weight and use it for the color assignment.

### Emotion prediction

EmoMTB   adopts Ekman’s ‘Big Six’ [39], i.e., an emotion model based on 6 basic emotions (happiness, sadness, anger, fear, disgust, surprise). From Ekman’s basic emotions, only happiness, sadness, anger, and fear are selected, since these are the ones typically used in previous works investigating musical emotions [40]. We use these 4 emotions to model both, users’ affect and songs’ emotions. Since emotional categories are more easily understandable by the general public than emotional dimensions, using the same categories to assess users’ and tracks’ emotions is considered the best compromise to ease the users’ cognitive load. However, datasets for music emotion recognition (MER) often adopt the dimensional model by Russel [41] or domain-specific models for musical emotions [42]. Thus, the lack of training data with songs and labels using the 4 chosen emotions necessitates the use of transfer learning for model training.

For this task, a multilayer perceptron classifier is trained on collections of social media and similar datasets that have been labeled according to the 4 chosen emotions [43]. Last.fm user-generated tags are used for predicting the songs’ emotions, while tweets are used to infer emotions of the ‘crowd’. OpenXBoW [44] is used to generate bag-of-words representations considering as input the emotional values from the lexica ANEW (Affective Norms for English Words [45]) and VADER (Valence Aware Dictionary and sEntiment Reasoner [46]).

### Recommending tracks

To retrieve the personalized recommendations from the Spotify API, EmoMTB   first fetches the top 5 short-term and long-term tracks of the user.^11^ Those tracks are then used as a seed to retrieve up to 200 recommendations.^12^ These recommendations are subsequently matched with the dataset of EmoMTB ,  where unavailable tracks are removed from the recommendations and available songs are mapped to their corresponding block within the landscape. The emotion-based re-ordering of the recommendation list leverages the confidence with which every emotion is predicted by the classifier for each track (Sect. 4.2). Thus, the tracks are sorted in descending order, e.g., if the user selects *happy* as their emotional state, the songs with the highest score for *happiness* will be shown on top of the list.

### Visualization and system architecture

We intentionally follow a lightweight interface design (e.g., not using texture-rich surfaces or features like particle emissions) to prevent users’ distractions from the music exploration experience, EmoMTB ’s  main purpose. The visualization is written in JavaScript and displayed via a browser using the three.js library^13^ for 3D landscape generation. Initially, we create a flat surface and a large sphere, which are used as floor and sky, respectively. While a concrete texture is assigned to the floor, the sky’s texture matches the currently selected emotion, as described in Sect. 3.4. A single directional light source acting as sun hovers far above the floor, adding additional reality to the scene. Its color, intensity, and the way shadows are cast again depend on the selected emotion. The colored blocks are placed and stacked throughout the landscape based on their previously determined coordinates (Sect. 4.1).

To navigate, a white hovering torus (Sect. 3.3), which sticks to the grid the blocks are placed on (to ease navigation), can be used to explore the world. When moving from block to block, smooth transition animations in the form of rapid initial movement and strong deceleration of the torus support the experience of a sticky grid. A perspective camera object, which provides the user with a third person view on the landscape, follows the torus by keeping it in the center area of its view.

*System architecture* The EmoMTB   system consists of three devices: a web server, the user’s smartphone, and the computer displaying the visualization. The web server provides both user-facing devices access to EmoMTB ’s  services, handles data storage (e.g., track data and their coordinates) and the connections to Spotify and Twitter. Moreover, the server acts as a relay to transfer information such as movement commands between phone and visualization. The main advantage of this approach is that any device can be used to run EmoMTB   (either visualization or control) by simply opening the corresponding web site, enabling easy deployment.

## Evaluation

We evaluated the different components of EmoMTB  regarding three aspects: clustering quality, accuracy of the emotion predictor, and user experience of the interface.

**Table 2:** Results of the emotion recognition experiments. Sample size, mean accuracy, recall, and precision across the 5 folds are given

Dataset	Size	Accuracy (%)	Recall (%)	Precision (%)
DailyDialogs [47]	618	43.8	44.4	44.5
Emotion-stimulus [48]	1,688	72.1	72.2	72.6
Emo-dataset-For-NLP [49]	9,592	71.2	71.2	72.4
Friends [50]	964	34.8	35.2	35.5
SemEval2007 [51]	356	36.2	36.2	35.5
SSEC [52][53]	252	21.0	20.0	20.3
TEC [54]	6,104	42.9	42.9	43.2
WASSA2017 [55]	2,012	65.6	65.6	65.8
Aggregated	21,480	59.0	59.1	59.2

### Quality of clustering

Previous approaches in audiovisual music interfaces usually qualitatively evaluate the homogeneity of the obtained clustering [5, 13, 14]. Following these studies, we also perform such a qualitative evaluation from a high-level point of view in Sect. 3.2; however, we also complement these visual assessments by providing a quantitative measure based on entropy inspired by Mayr [56] and Vad et al. [10]. To assess the genre homogeneity of the clustering, we investigate the local genre distributions of the tracks among the entire landscape. In particular, given the tiled map of EmoMTB ,  we slide a 3$\times$3-tile window with stride 3 over the whole map and examine the genre distribution within the window, effectively forming small clusters. We compute the genre entropy within the window as a proxy for its inhomogeneity as:

$$H(w) - \sum _g \frac{t^g_w}{t_w} \cdot log\frac{t^g_w}{t_w}$$

where $t^g_w$ represents the number of tracks in window *w* that belong to genre *g*, while $t_w$ is the total number of tracks in *w*. We then aggregate the entropy values for all windows and compute the total genre entropy of the landscape as:

$$H(\Omega ) = \sum _w H(w) \frac{t_w}{t}$$

where *t* represents the total number of tracks. The total genre entropy equals 0 if each window encloses only the tracks of a specific genre, indicating the most coherent clustering, while the entropy reaches its maximum ($log(12) \approx 2.485$)^14^ when each genre has an equal chance to appear in any given window. The total genre entropy of EmoMTB   is 0.168, which represents only $6.7\%$ of the maximum entropy and indicates a high genre coherency within the clusters. For further comparison, we randomly shuffle the genres among the tracks while keeping the tracks’ positions fixed and compute the genre entropy of this new landscape. We repeat this random shuffling 5 times resulting in a genre entropy of $1.241 \pm 0.001$, which accounts for circa $50\%$ of the maximum entropy. We, therefore, conclude that EmoMTB ’s  positioning of tracks on the landscape results in highly homogeneous music clusters in terms of genres.

### Emotion recognition performance

The main difficulty in training and evaluating a model to identify emotion in songs is that accessible datasets tend to be small in size. In addition, the task of finding a suitable dataset becomes even more challenging when considering basic emotions, as typically MER datasets adopt other models (Sect. 4.2). Therefore, the chosen method relies on transfer learning: A model is trained and tested on Twitter corpora for sentiment analysis and then used to classify emotions from Last.fm tags.

The database used for training and testing is composed of 8 individual datasets, which after being aggregated and cleaned, contain a total of 21,480 samples. In a preprocessing phase, each dataset is cleaned. This involves deleting samples with labels other than the 4 emotional categories used by EmoMTB ,  selecting samples with unambiguous labels (some datasets have multiple labels per sample) and applying a uniform labeling convention (e.g., changing ‘joy’ to ‘happiness’ or ‘sad’ to ‘sadness’). Furthermore, each individual dataset is downsampled in order to guarantee a balanced distribution across the 4 emotional labels.

A joint classification model is then trained on this aggregated dataset,^15^ and evaluated following a fivefold cross-validation setup with Monte Carlo sampling, i.e., for each fold, the test set and the validation set comprise 20% of randomly selected samples each. In Table 2, classification results on the test set (mean accuracy, recall, and precision) across the 5 folds, are given for each individual dataset as well as the aggregated one. The results show, as expected, that the model performs better for larger datasets, which (being larger) had a more prominent role during training, thus positively influencing the model’s classification performance.

### Qualitative evaluation of the interface

**Fig. 7:**
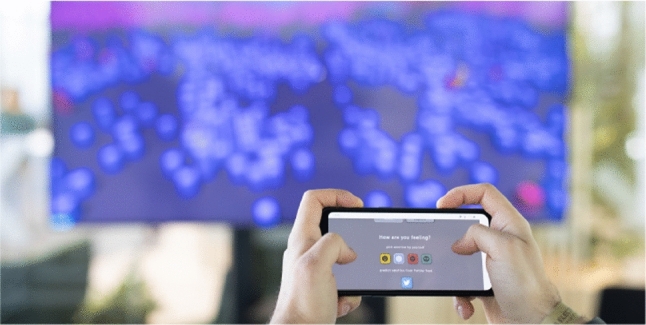
Impression of EmoMTB   during the Ars Electronica Festival 2021. Credit: digital media arts festival

EmoMTB   was presented to the general public at the Ars Electronica Festival 2021,^16^ one of the biggest media arts festivals. An impression of the exhibit’s setup can be obtained from Fig. 7. The exhibit took place in a glass cube (approximately 5 $\times$ 5 m). The big screen showing the landscape was positioned on one side of the cube, the visitors stood at a distance of about 3 m away from it and used their smartphones to interact with the city of music.

We leveraged the opportunity provided by the festival to conduct a qualitative evaluation of EmoMTB .  In particular, we follow the framework of Knijnenburg et al. [57] and assess the system-related dimension of the user experience. During the exhibit, visitors could tell us their email addresses, which we used to invite them to take part in a follow-up online questionnaire. The questionnaire was composed of open questions and aimed to obtain qualitative feedback on different aspects of EmoMTB .  More precisely, we asked participants the following questions and provided free-form text fields for their answers, using Google Forms.^17^

- Which is the aspect, such as the entertainment or the possibility to discover new music, that you consider unique and most relevant from your experience while interacting with EmoMTB ? 
- How would you describe the attractiveness/visual appeal of the landscape?
- How would you describe the usage of EmoMTB   interface in terms of complexity?
- To which extent do you think that using the metaphor of the city as a way to explore music collections is appropriate and which alternative solutions could you imagine for such a purpose?
- How was your impression of the emotional component of EmoMTB ,  for instance, concerning the different themes of the landscape and the emojis related to each track?
- To which extent were the recommendations you received satisfactory?
- How would you describe your overall experience?

Participation was anonymous. While only 8 users participated in the survey, we received highly interesting qualitative feedback, summarized in the following.

Concerning the most relevant aspect of EmoMTB ,  the majority of participants (6) highlighted that discovering new songs was the most useful and interesting feature; 2 also mentioned the importance of the entertainment and visual components as unique and very original aspects. Most of the users found the visual appeal of the landscape good (6), but also rather simple (4); indeed, 2 participants mentioned that the landscape would benefit of additional elements, such as trees. Similarly, most of the participants (6) also agreed on the simplicity of the interface, whose functionality was easy to understand and intuitive to use; still, 2 users also mentioned that the functionality, although simple, might not be so straightforward for people unfamiliar with mobile phone games.

Concerning the quality of recommendations, the participants were generally satisfied (3) or very satisfied (3); besides their quality, 2 users expressed that the recommendations were simply a (great) starting point to freely explore the landscape. The most critical aspect, from the participants’ point of view, was the emotional component. Although a majority (5) considered the emotional themes very appropriate, and generally the emotional component interesting and inspiring (3), some users (4) also indicated that this part could be improved, since the emojis associated with the tracks did not always match their perceived emotion.

Despite the limitations, the participants unanimously agreed on the appropriateness of the city metaphor and described their general experience as very positive, highlighting the role of the entertainment and enjoyable components. As for suggestions for further developments, 2 participants suggested building a universe or a music shop as alternative topics to inspire future landscapes. In addition, 1 participant suggested that creating emotional clusters, i.e., changing the landscape itself (besides the sky) according to the emotional themes, would be a very useful feature in order to enhance the emotional experience.

## Conclusions and future work

*Summary and Impact.* While modern music recommender systems achieve remarkable results by employing algorithm-driven approaches, they may often dissatisfy users due to their internal biases and limitations with respect to the presentation of results (by resorting to lists as a means of showing results). In this work, we put the user back in the loop, allowing them to enhance their listening experience and fostering their curiosity and intuition.

For this purpose, we present EmoMTB ,  an immersive audiovisual interface that integrates precision of algorithmic recommendation with serendipity and excitement of free browsing in a single experience. The recommendation part of the system allows users to quickly find a starting point for their music journey, while the exploration part helps them escape their filter bubble and encounter new enjoyable tracks they would not be able to find otherwise. The entire music collection of almost half a million tracks is laid out in front of the user in a city-like landscape. The proximity of every two tracks corresponds to their similarity in terms of genre and audio features. This creates a space of continuous music genre transition. Given a number of initial recommendations as landmarks, the user is able to instantly travel to one of them and start exploring nearby tracks, smoothly transitioning to related music styles or genres. The landmarks are recommended personally to each user and are re-ranked based on their emotional state.

Offering this outstanding combination of features, we believe EmoMTB   has the potential to impact the next generation of music players. While still being a prototype, EmoMTB ’s  ability to offer its users new experiences and encounters, in particular related to the diversification of their music knowledge and taste, is likely to attract music aficionados and indulge occasional listeners alike.

We evaluated various aspects of EmoMTB ,  in particular the genre homogeneity of nearby tracks in the virtual city and the performance of the emotion recognizer. We also conducted a qualitative user study by means of a web-based post-experience questionnaire, in which participants of a media arts festival who tried EmoMTB   provided valuable feedback.

*Limitations* Even if EmoMTB   received highly positive feedback from the hundreds of people at the Ars Electronica Festival 2021, few limitations have been pointed out. First, the current version requires its users to have a Spotify account, because of technical and legal reasons. Second, the performance of the emotion recognizer is limited, and the integration of emotion-awareness into the interface is not very sophisticated. Third, the user requires two screens to enjoy the full experience, i.e., a small screen (commonly a smartphone) for interacting with the landscape that is shown on a big screen to ensure an immersive music exploration experience.

*Future work* Next to addressing the limitations outlined above, we contemplate additional directions for further research and development of EmoMTB .  First, its interaction capabilities could be extended, allowing users to modify the landscape, or even to create their own individual cities. Second, the visualization could be made more lively by adopting additional metaphors, e.g., tramways that represent curated or automatically created playlists. Third, the single-user-mode could be enhanced to a multi-user-experience, with different avatars representing different users. Thereby, exploring a music collection could be turned into a truly collaborative experience. Finally, since popularity biases are one major drawback of today’s music recommendation engines and EmoMTB   provides a remedy by granting all tracks equal exposure (they are all included in the visualization irrespective of their popularity), more research should be investigated into the mitigation of such biases by means of intelligent music discovery interfaces.

## Data Availability

The datasets used in the studies are available on http://www.cp.jku.at/datasets/LFM-2b.

## Footnotes

1 https://newsroom.spotify.com/company-info.

2 https://www.deezer.com/en/company/press.

3 https://www.amazon.com/b?ie=UTF8 &node=15730321011.

4 https://www.allmusic.com.

5 https://www.last.fm.

6 https://developer.spotify.com/documentation/web-api/.

7 http://www.cp.jku.at/datasets/LFM-2b/.

8 https://developer.spotify.com/documentation/web-api/reference/#/operations/search.

9 https://developer.spotify.com/documentation/web-api/reference/#/operations/get-several-audio-features.

10 https://everynoise.com/.

11 https://developer.spotify.com/documentation/web-api/reference/#/operations/get-users-top-artists-and-tracks.

12 https://developer.spotify.com/documentation/web-api/reference/#/operations/get-recommendations.

13 https://github.com/mrdoob/three.js/.

14 By the Jensen inequality, $H(X) = E[log(\frac{1}{p(X)})] \le log(E[\frac{1}{p(X)}]) = log(n)$ where *n* is the number of variable’s outcomes.

15 Note that by using a model pre-trained on the aggregated dataset we aim to generalize its usability, for both emotion recognition tasks, i.e., predicting emotions from tags and from tweets.

16 https://ars.electronica.art/newdigitaldeal/en.

17 https://www.google.com/forms/about.
